# Cancer screening and prevention education in standardized training residents: A cross-sectional survey in single center

**DOI:** 10.1186/s12909-022-03876-9

**Published:** 2022-12-01

**Authors:** Juntao Ran, Ziying Dai, Song Wang, Li Li, Ya Zheng, Guofeng Qu, Chun Liu, Ming Chen

**Affiliations:** 1 Department of Radiation Oncology, the First Hospital of Lanzhou University, Lanzhou, 730000 People’s Republic of China; 2 Office of Standardized Training for Physicians, The first Hospital of Lanzhou University, Lanzhou, China; 3 Department of Gastroenterology, The first Hospital of Lanzhou University, Lanzhou, China; 4 Department of Nuclear Medicine, The first Hospital of Lanzhou University, Lanzhou, China

**Keywords:** Residents, Standardized training, Tumor, Prevention and screening, Cross-sectional study

## Abstract

**Background:**

Standardized residency training is an essential aspect of enhancing the ability of cancer prevention and screening of residents. The current study was performed to investigate tumor prevention, screening literacy and the training demands of standardized training residents and explore related influencing factors.

**Methods:**

A cross-sectional survey was conducted among 320 residents of The First Hospital of Lanzhou University. An online, self-designed questionnaire was employed to investigate tumor prevention and screening, training status, and the requirements of residents. Data were analyzed using Fisher’s exact test.

**Results:**

The mean age of the 320 participants was 26.04 ± 1.85 years;133, 83, and 104 were in the 1st, 2nd and 3rd year of standardized training, respectively. Among the common carcinogenic factors, smoking, infectious agents, and drinking were more correlated with tumors by 72.19, 66.57, and 64.38% of the physicians, respectively. Excess body weight, an insufficient intake of fruits and dietary fiber, and a lack of exercise were correlated with tumors by only 26.56, 25, and 23.44% of the physicians, respectively. The proportion of physicians providing an accurate answer to the tumor screening question ranged from 23.13 to 93.13%. The lowest accuracy was 23.13% for the initial age of regular breast cancer screening in general-risk women. The maximum rate of the primary liver cancer screening methods was 93.13%. Postgraduates and residents of oncology practitioners considered excess body weight and the insufficient intake of fruits and dietary fiber more relevant to cancer (*P* < 0.05). Male residents viewed more associations between tumors and a lack of exercise and air pollution (*P* < 0.05). Overall, 71.26% of participants felt that their tumor prevention and screening knowledge was poor and 95.31% thought they needed standardized tumor prevention and screening training.

**Conclusion:**

Tumor prevention and screening literacy of standardized training residents should be further improved. There is a huge knowledge demand for tumor prevention and screening. Therefore, it is vital to build a training program in line with the requirements of cancer prevention and control efforts that focus on improving literacy among residents.

## Introduction

Malignancies are a serious threat to public health with an estimated 4.82 million new malignant tumors and 3.21 million deaths in China in 2022 [1]. Worryingly, tumor morbidity continues to rise; 45.2% of tumor-related deaths are primarily caused by preventable carcinogenic factors in China [2]. Early detection and treatment are the most effective approaches to reduce tumor morbidity and mortality [3]. Therefore, it is of vital importance to perform tumor prevention and screening protocols. The Healthy China Action [4] Cancer Prevention and Control Implementation Plan (2019-2022) is dedicated to promoting early detection and raising awareness of cancer prevention. Due to the low level of systematic awareness of cancer risk factors and the lack of awareness and ability of self-health management, there is an urgent need to establish innovative systems for the primary prevention of cancer at both population and individual level [5]. Improving the literacy of standardized training residents for providing expert recommendations to the public are significant strategies that can enhance cancer prevention and screening efforts. All medical clinicians with bachelors degree and above will receive standardized training as residents in China. This programme aims to help residents reach the same professional standards during a 3 year training period and involves several types of examinations during the different phases of training to ensure the quality of residents. The standardized training of residents is well implemented and resident physicians enjoy an improvement in clinical ability [6]. However, the training emphasizes disease diagnosis and treatment in the present stage, while efforts related to cancer prevention and screening is minimal. The literature on cancer prevention and screening knowledge in standardized training residents in China is scarce, furthermore, the available evidence among medical workers focusing on lung [7] and cervical cancer [8] screening demonstrates that the knowledge needs to be improved. In this study, we aimed to target residents and analyze their literacy with regards to cancer prevention and screening knowledge, to investigate the training demands of residents and provide reference guidelines for the establishment of novel and targeted programs.

## Methods

### Survey procedures

This cross-sectional survey involved standardized training residents who underwent standardized training in The First Hospital of Lanzhou University in February 2021. The survey was launched in The Questionnaire Star, an online professional questionnaire survey platform in February 2021. The platform link was pushed to a corresponding WeChat group, and a reminder of the link was issued regularly to encourage respondents to complete the questionnaire. The residents independently completed the questionnaire, and the researchers supplied the necessary guidance and instruction. All surveys were retrieved in March 2021 and the researchers collected appropriate data.

### Questionnaire

An online questionnaire was used to collect data using the “Residents Cancer Prevention and Screening Literacy Questionnaire” prepared by the study team. The questionnaire was based mainly on common carcinogenic factors, tumor screening guidelines and consensus in China [2]. This involved seven cancers: breast, lung, gastric, esophageal, liver, colorectal, and cervical [9–15]. The questionnaire featured an independent design that was combined with the study goal and content and was reviewed by experts in preventive medicine and statistics. The questionnaire consisted of three modules: prevention knowledge module including 9 questions surrounding common carcinogenic factors, screening knowledge module with 13 questions focusing on initial screening age and screening methods of seven cancers and training status and demands module with 11 questions surrounding the current status and demand of education, as summarized in Table 1. In addition, a presurvey was undertaken to check whether the questionnaire had ambiguities and a reasonable network procedure. After correcting the flaws, a standard version was completed, with a Cronbach’s alpha coefficient of 0.868.

**Table 1:** Questionnaire contents

Modules	Contents
Prevention knowledge (9 questions)	1. Excess body weight 2. Insufficient intake of fruits and dietary fiber 3. Lack of exercise 4. Smoking 5. Drinking 6. Consumption of red meat, processed meat and cured foods 7. Air pollution (PM_2.5_) 8. Second-hand smoke 9. Infectious agents (HP/HBV/HCV/HPV/EBV/HIV/HHV-8)
Screening knowledge (13 questions)	10. Initial age of regular breast cancer screening in general-risk women 11. Primary breast cancer screening methods for general-risk women 12. To what extent can breast cancer mortality be reduced overall by mammography screening? 13. Primary lung cancer screening methods 14. Lung cancer screening intervals 15. To what extent can cancer mortality be reduced by low-dose CT screening in high-risk individuals? 16. Initial screening age for esophageal cancer in high-risk individuals 17. Initial screening age for gastric cancer 18. Liver cancer screening intervals for high-risk individuals 19. Primary liver cancer screening methods 20. Primary cervical cancer screening methods 21. Initial age for high-risk individuals undergoing colonoscopy 22. Primary colorectal cancer screening methods for high-risk individuals
Training status and demands (11 questions)	23. Is there a specialized training option for tumor prevention and screening scheduled during the training period? 24. Does the department teach tumor prevention and screening knowledge during the training period? 25. Is the training in tumor prevention and screening sufficient during the training period? 26. Do you have adequate knowledge reserve to provide tumor prevention and screening services? 27. Do you think a standardized training modality is needed for tumor prevention and screening? 28. A reasonable training time 29. The best training way 30. The importance of common tumors epidemiology in the training content 31. The importance of evaluation criteria of common tumors for high-risk individuals in the training content 32. The importance of primary screening techniques for common tumors in the training content 33. The importance of common tumor prognosis in the training content

### Investigation contents and methods

The main contents of investigation were divided into three aspects: tumor associations with common carcinogenic factors, tumor screening knowledge, and the current situation and training demands for tumor prevention and screening. The content relating to tumor associations with common carcinogenic factors was focused on whether residents had mastered common carcinogenic factors and knew the factors involved in tumorigenesis. There were nine questions; the ‘not at all’, ‘minimally’ and ‘somewhat’ options provided evidence for a low correlation between carcinogenic factors and tumors. In contrast, the ‘moderately’ and ‘very confident’ provided evidence of a strong correlation between factors and tumors. The content related to tumor screening knowledge focusses on the extent to which residents had mastered tumor screening knowledge; there was 13 questions and four options with only one correct answer. Finally, we set 11 questions to focus on training status and demand; the options were ‘yes’, ‘no’ and ‘unsure’. The options provided for reasonable training time were < 1 course, 1 to 5 courses and > 10 courses. The options for optimal training modalities were lectures, problem-based learning (PBL), *case*-based learning (CBL), and others. The options for training content were not important, important, very important and unsure.

### Statistical analysis

STATA 16.0 (StataCorp LLC, College Station, TX, USA) was employed for data analysis. Measurement data conforming to the normal distribution or an approximate normal distribution were expressed as mean and standard deviation. Numerical data were reported as frequency and percentage [case (%)], and Fisher’s exact test was used to compare data between the groups. *P* values < 0.05 were considered statistically significant.

## Results

### Subject characteristics

The demographic characteristics of the standardized training residents are shown in Table 2. A total of 320 residents were included: 100 males (31.25%) and 220 females (68.75%). In total, 133, 83, and 104 residents were in the first, second, and third years of standardized residency training, respectively. Of the 320 residents, 145 were in undergraduate education and 175 were in postgraduate education; 51 focused on oncology and 269 focused on other areas. Before the questionnaires were administered, each subject was informed of the study content, and all participants provided signed and informed consent. In total, 320 questionnaires were distributed and 320 valid copies were returned, with a valid recovery rate of 100%.

**Table 2:** General characteristics of the survey residents (*n* = 320)

Characteristic	[n(%)]
Gender	
Male	100(31.3)
Female	220(68.7)
Education	
Undergraduate	145(45.3)
Postgraduate	175(54.7)
Year in residency training	
Year 1	133(41.6)
Year 2	83(25.9)
Year 3	104(32.5)
Area of current training	
Focused in oncology	51(15.9)
Focused in other area	269(84.1)

### Cancer prevention knowledge

**Fig. 1:**
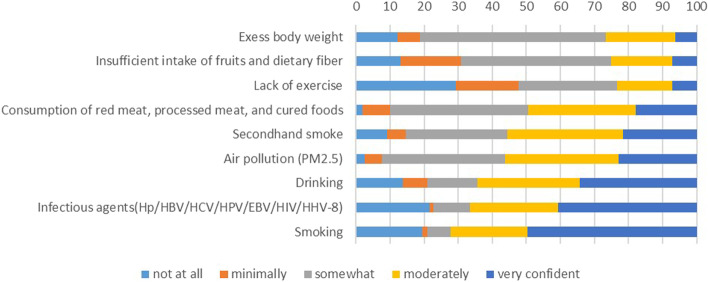
Assessment of tumor associations with common carcinogenic factors

With regards to common carcinogenic factors, 72.19% (231), 66.57% (213), and 64.38% (206) of the recruited participants considered smoking, infectious factors, and drinking to be highly associated with tumors, respectively. In contrast, only 26.56% (85), 25% (80), and 23.44% (75) of the total number of subjects thought excessive weight, an insufficient intake of fruits and dietary fiber, and a lack of exercise were closely related to malignancies, as detailed in Fig. 1.

### Tumor screening knowledge

**Fig. 2:**
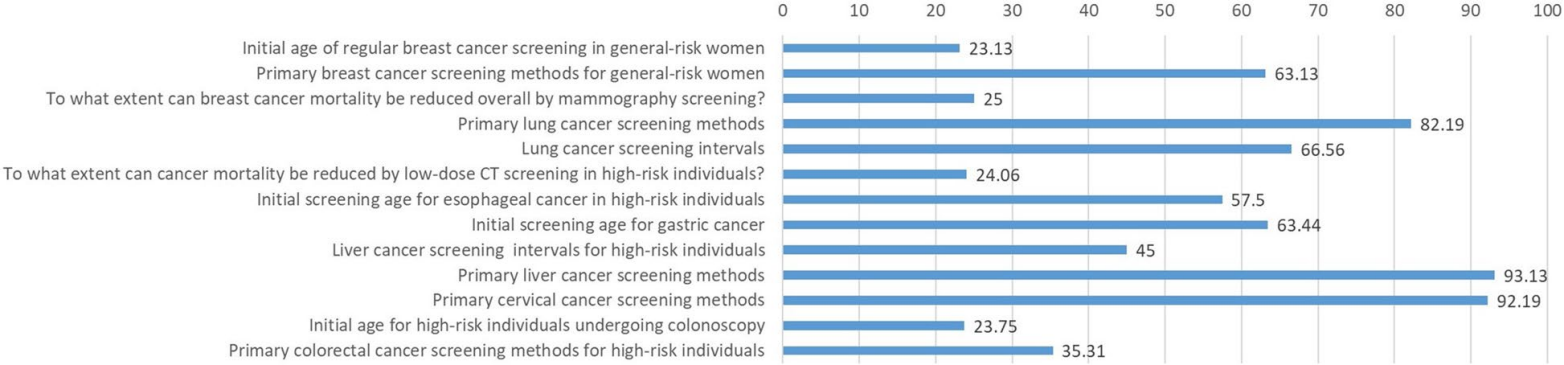
Proportions of correct answers for the tumor screening questions

The proportion of accurate answers to the 13 questions designed for the seven cancer screening factors ranged from 23.13 to 93.13%; the lowest proportion of 23.13% was related to the initial age for regular breast cancer screening in general-risk women while the highest proportion of 93.13% related to primary liver cancer screening methods. The proportion of accurate answers for the initial age (age range) for tumor screening and the role of standardized screening techniques in reducing specific tumor mortality were both low. In contrast, the proportion of accurate answers for standardized screening techniques and methods were high for other malignancies, as illustrated in Fig. 2.

### Analysis of factors related to tumor prevention and screening literacy among residents with varied characteristics

Subgroup analysis for tumor prevention and screening literacy showed that residents with postgraduate education and an oncology specialism considered excess body weight to be associated with tumors (*P* < 0.05). Residents with postgraduate education considered insufficient dietary fiber intake to be closely related to cancer (*P* < 0.05). Male residents reported that a lack of exercise and air pollution were associated with malignancy (*P* < 0.05). Of the cancer screening questions, residents with postgraduate education showed superior accuracy in answering the question relating to primary liver cancer screening methods (*P* < 0.05). The residency period was associated with proportion of correct answers relating to the extent by which breast cancer mortality can be reduced overall by mammography screening (*P* < 0.05). Residents with oncology specialism had a better proportion of correct answers with regards to primary lung cancer screening methods (*P* < 0.05), as indicated in Table 3.

**Table 3:** Status of current cancer prevention and screening knowledge in standardized training residents [N(%)]

Question	Gender		*P*	Education		*P*	Year in residency training			*P*	Area of current training		*P*
	Female (*n* = 220)	Male (*n* = 100)		Undergraduate (*n* = 145)	Postgraduate (*n* = 175)		Year 1 (*n* = 133)	Year 2 (*n* = 83)	Year 3 (*n* = 104)		Focused in other areas (*n* = 269)	Focused in oncology (*n* = 51)	
Excess body weight													
low correlation	163 (74.1)	72 (72.0)	0.685	121 (83.4)	114 (65.1)	< 0.001	94 (70.7)	63 (75.9)	78 (75.0)	0.641	207 (77.0)	28 (54.9)	0.002
strong correlation	57 (25.9)	28 (28.0)		24 (16.6)	61 (34.9)		39 (29.3)	20 (24.1)	26 (25.0)		62 (23.0)	23 (45.1)	
Insufficient intake of fruits and dietary fiber													
low correlation	169 (76.8)	71 (71.0)	0.269	117 (80.7)	123 (70.3)	0.038	100 (75.2)	61 (73.5)	79 (76.0)	0.932	202 (75.1)	38 (74.5)	1.000
strong correlation	51 (23.2)	29 (29.0)		28 (19.3)	52 (29.7)		33 (24.8)	22 (26.5)	25 (24.0)		67 (24.9)	13 (25.5)	
Lack of exercise													
low correlation	177 (80.4)	68 (68.0)	0.022	114 (78.6)	131 (74.9)	0.508	105 (78.9)	61 (73.5)	79 (76.0)	0.653	207 (76.9)	38 (74.5)	0.720
strong correlation	43 (19.6)	32 (32.0)		31 (21.4)	44 (25.1)		28 (21.1)	22 (26.5)	25 (24.0)		62 (23.1)	13 (25.5)	
Air pollution (PM2.5)													
low correlation	107 (48.6)	33 (33.0)	0.011	62 (42.8)	78 (44.6)	0.821	60 (45.1)	33 (39.8)	47 (45.2)	0.709	116 (43.1)	24 (47.1)	0.646
strong correlation	113 (51.4)	67 (67.0)		83 (57.2)	97 (55.4)		73 (54.9)	50 (60.2)	57 (54.8)		153 (56.9)	27 (52.9)	
Primary liver cancer screening methods													
Right	206 (93.6)	92 (92.0)	0.636	130 (89.7)	168 (96.0)	0.028	123 (92.5)	77 (92.8)	98 (94.2)	0.886	249 (92.6)	49 (96.1)	0.548
Wrong	14 (6.4)	8 (8.0)		15 (10.3)	7 (4.0)		10 (7.5)	6 (7.2)	6 (5.8)		20 (7.4)	2 (3.9)	
To what extent can breast cancer mortality be reduced overall by mammography screening?													
Right	50 (22.7)	30 (30.0)	0.167	40 (27.6)	40 (22.9)	0.365	40 (30.1)	23 (27.7)	17 (16.3)	0.036	68 (25.3)	12 (23.5)	0.862
Wrong	170 (77.3)	70 (70.0)		105 (72.4)	135 (77.1)		93 (69.9)	60 (72.3)	87 (83.7)		201 (74.7)	39 (76.5)	
Primary lung cancer screening methods													
Right	186 (84.5)	77 (77.0)	0.116	115 (79.3)	148 (84.6)	0.242	105 (78.9)	74 (89.2)	84 (80.8)	0.144	216 (80.3)	47 (92.2)	0.046
Wrong	34 (15.5)	23 (23.0)		30 (20.7)	27 (15.4)		28 (21.1)	9 (10.8)	20 (19.2)		53 (19.7)	4(7.8)	

### Training status and demand for cancer prevention and screening

During standardized training, it was evident that there was existing knowledge relating to tumor prevention and screening, accounting for 28.13% (90) of the total number of residents; 58.13% (186) and 13.75% (44) of the residents said they had ‘no’ training or were ‘unsure’, respectively. Overall, 56.88% (182) of the total number of residents thought departments taught tumor prevention and screening; 26.88% (86) and 16.25% (52) of the residents replied ‘no’ or were ‘unsure’, respectively. Insufficient and adequate tumor prevention and screening training accounted for 69.37% (222) and 30.63% (98) of the total residents, respectively. Regarding training demand, 71.26% (228) of the residents believed that their cancer prevention and screening knowledge was insufficient, and standardized training was noted by 95.31% (305) of the residents. Analysis showed that 35.63% (114) and 50.63% (162) of the residents considered lectures and CBL instruction the best methods, respectively, while 56.88% (182) believed that one to five training sessions was the most optimal training format. Training content, including common tumor epidemiology, screening criteria for high-risk groups, screening techniques, and tumor prognosis, were considered highly significant by residents with a minimum requirement of greater than 95%.

## Discussion

The target population for existing investigations relating to the cognitive level of cancer prevention and screening is predominantly ordinary Chinese inhabitants [16]. There are limited surveys relating to residents undergoing standardized training. Understanding and raising the literacy of residents can effectively optimize and improve cancer prevention and control services. This study investigated the literacy of residents from two dimensions: tumor associations with common carcinogenic factors and tumor screening literacy. We also investigated the training status and relevant demands of the residents. The information obtained from this survey showed that residents had good knowledge of cancer prevention and screening. However, there were shortcomings in some points, and appropriate training still needs to be strengthened. Several studies have reported that oncology teaching in undergraduate medical education and postgraduate residency training is insufficient [17, 18]. The present study indicated distinct cognitive dissonance among residents regarding the correlation between common carcinogenic variables and tumors. Most residents believed that smoking, drinking, infectious factors, air pollution, and secondhand smoke were closely related to tumorigenesis. In contrast, only 26.56, 25, and 23.44% of the residents believed that excessive body weight, an insufficient intake of fruits and dietary fiber, and a lack of exercise were associated with tumor development, respectively. This situation may relate to the fact that current postgraduate training education opportunities to enhance knowledge in cancer prevention are limited [19]. This also might be attributed to incomplete training during the normal study period. Normal instruction was prone to highlight carcinogenic factors with high cognition and ignore poorly understood carcinogenic factors, thus resulting in the absence of teaching relating to the associations between tumors and obesity, a lack of exercise, and an insufficient intake of dietary fiber. There is clear evidence for the close relationship between obesity, exercise and tumors [20, 21]. Therefore, during the training stage, it is vital to reinforce formal training to improve the acceptance and awareness of residents with regards to various carcinogenic factors and provide patients with the most reasonable and comprehensive suggestions. Our research found that the residents had high levels of knowledge with regards to tumor screening technology. However, there were insufficient perceptions regarding the initial screening time and the value of tumor screening, with more emphasis on technology than prevention. Moreover, the present training period appears to focus mainly on disease diagnosis and treatment skills and only provides a limited amount of information relating to systematic training in cancer screening. Cancer screening plays a definite role in improving the early detection rate and reducing tumor mortality [22, 23]. Therefore, standardized teaching should reinforce information regarding the initial screening age and the value of screening for high-risk individuals, promote the initiative of residents with regards to cancer screening services, and improve active awareness during treatment in medical institutions.

In addition, our results demonstrated that gender, education level, residential training duration, and oncology specialism were the primary factors influencing the literacy of cancer prevention and screening literacy in residents. Highly educated and oncology specialty residents had been exposed to more information relating to cancer prevention and control during standardized training, thus improving their grasp of common carcinogenic factors and screening skills. Deeper engagement in exercise and more focus on the harmful consequences of air pollution might be the critical reasons for the high awareness of male residents with regards to the association between a lack of exercise, air pollution and tumor growth. In addition, residential training duration was inversely correlated with the proportion of accurate answers for the question asking to what extent breast cancer mortality might be reduced overall by mammography screening, with the maximum proportion of accurate answers given by physicians in the first training year. However, the overall proportion of correct answers was merely 25%; this was due to the lack of systematic education in different grades and the intersection of turnaround time for the residents.

The results of our study relating to the current training status of residents showed that only 28.13% of the residents received professional cancer prevention and screening training, and that most residents still relied on spontaneous teaching. A study conducted by Cheung et al. found that only 12.5% residents reported more than 1 week of cancer education in their training program and 75% indicated that 1 to 5% of their entire curriculum focused on cancer [17]. Furthermore, 69.37 and 71.26% of the residents believed that their training related to cancer prevention and screening, and their relevant knowledge was insufficient, respectively. Furthermore, the survey revealed that one to five training courses were a feasible modality based on lectures and CBL teaching. Strengthening the oncological knowledge of residents [24] and the specialized knowledge of oncological physicians [25] could increase their ability to prevent and control cancer. The prevention of primary and secondary tumors, along with secondary prevention, is essential approaches that can reduce the burden of tumor-related diseases in China [26, 27]. Therefore, residents undergoing standardized training are deemed to require a pivotal window to promote cancer prevention and control and help guide the implementation of public health projects. Training in cancer prevention and screening is an important safeguard to improve the literacy of physicians and the level of cancer prevention and control efforts. However, there is still a significant lack of systematic and standardized training. Therefore, we recommend that experience in oncology departments be arranged for at least one to 2 months during the training period to construct a residency training program that meets the requirements for tumor control and prevention. Furthermore, teachers are required to carry out extensive instruction on cancer prevention and screening to ensure the continuous improvement of literacy in residents.

Several limitations should be considered when interpreting these results. First, the residents were based in a single hospital, this may have caused bias. Therefore, a large multi-center survey is needed to further confirm our results. Secondly, we did not investigate the attitudes and other opinions or intentional behavior of individuals. Thus, our results mainly demonstrate the level of knowledge mastery. Although knowledges relating to cancer prevention and screening represents a basic foundation, attitudes and behavior are also very important. Here, we conducted an ongoing multi-center cross-sectional survey of the attitudes and clinical behavior of residents undergoing standardized training.

In summary, residents with standardized training have an excellent grasp of tumor prevention and screening, although there is still space for improvement. However, training relating to cancer prevention and screening is clearly lacking within the standardized training stage; a systematic and standardized scheme needs to be developed. Therefore, extending the existing training program and integrating cancer prevention and control knowledge into regular instruction will help to improve the literacy of residents and promote cancer prevention and control.

## Data Availability

The datasets generated or analyzed during the current study are available from the corresponding author on reasonable request.

## Abbreviations

PBL: Problem-based learning
CBL: *Case*-based learning
